# The value of repeat patient testing for SARS-CoV-2: real-world experience during the first wave

**DOI:** 10.1099/acmi.0.000239

**Published:** 2021-07-08

**Authors:** Alex Zhu, Margaret Creagh, Chao Qi, Shannon Galvin, Maureen Bolon, Teresa Zembower

**Affiliations:** ^1^​ Northwestern University, Evanston, Illinois, USA; ^2^​ Analytics, Northwestern Healthcare, Chicago, Illinois, USA; ^3^​ Clinical Microbiology Laboratory, Northwestern Memorial Hospital, Northwestern Healthcare, Chicago, Illinois, USA; ^4^​ Department of Pathology, Northwestern University Feinberg School of Medicine, Chicago, IL, USA; ^5^​ Department of Medicine, Division of Infectious Diseases, Northwestern University Feinberg School of Medicine, Chicago, IL, USA

**Keywords:** SARS-CoV-2, repeat testing, COVID-19 laboratory diagnosis

## Abstract

**Introduction:**

Reports of false-negative quantitative reverse transcription PCR (RT-qPCR) results from patients with high clinical suspension for coronavirus disease 2019 (COVID-19), suggested that a negative result produced by a nucleic acid amplification assays (NAAs) did not always exclude the possibility of COVID-19 infection. Repeat testing has been used by clinicians as a strategy in an to attempt to improve laboratory diagnosis of COVID-19 and overcome false-negative results in particular.

**Aim:**

To investigate whether repeat testing is helpful for overcoming false-negative results.

**Methods:**

We retrospectively reviewed our experience with severe acute respiratory syndrome coronavirus 2 (SARS-CoV-2) testing, focusing on the yield of repeat patient testing for improving SARS-CoV-2 detection by NAA.

**Results:**

We found that the yield from using repeat testing to identify false-negative patients was low. When the first test produced a negative result, only 6 % of patients tested positive by the second test. The yield decreased to 1.7 and then 0 % after the third and fourth tests, respectively. When comparing the results produced by three assays, the Centers for Disease Control and Prevention (CDC) SARS CoV-2 RT-qPCR panel, Xpert Xpress CoV-2 and ID NOW COVID-19, the ID NOW assay was associated with the highest number of patients who tested negative initially but positive on repeat testing. The CDC SARS CoV-2 RT-qPCR panel produced the highest number of indeterminate results. Repeat testing resolved more than 90 % of indeterminate/invalid results.

**Conclusions:**

The yield from using repeat testing to identify false-negative patients was low. Repeat testing was best used for resolving indeterminate/invalid results.

## Introduction

Rapid detection of severe acute respiratory syndrome coronavirus 2 (SARS-CoV-2), the aetiological agent associated with coronavirus disease 2019 (COVID-19), is critical for infection control and patient management. Detection of viral RNA with nucleic acid amplification (NAA) has high sensitivity *in vitro* and provides rapid results. Currently, established procedures for nucleic acid tests for SARS-CoV-2 include quantitative reverse transcription PCR (RT-qPCR) and isothermal amplification methods. The Centers for Disease Control and Prevention (CDC) RT-qPCR panel was the first assay implemented in the USA. The panel targeted the nucleocapsid protein (N) gene of SARS-CoV-2 and was reported to have an analytical limit of detection of five copies/reaction of quantified RNA transcripts [1]. Later, many commercial assays received emergency use authorization (EUA) from the Food and Drug Administration (FDA) and were quickly adopted by clinical laboratories.

Reports of false-negative RT-qPCR results from patients with high clinical suspension for COVID-19 suggested that a negative result produced by a NAA assay did not exclude the possibility of COVID-19 infection [2, 3]. Several possible reasons for false-negative results have been proposed. Inadequate sampling and timing of testing during the disease process are the primary reasons for false-negative results [4]. In these situations, the viral RNA may be below the limit of detection of the test. Genetic diversity and rapid evolution of SARS-CoV-19 have been observed [5, 6]. Sequence variability can cause mismatch between the primers and probes and the targets, which may reduce assay performance and result in false-negative results. Strategies such as multiple target amplification were used to improve assay performance. False-negative results may also be a result of poor analytical performance of the molecular tests used. Repeat testing has been used by clinicians as a strategy to improve laboratory diagnosis of COVID-19 and overcome false-negative results in particular. In addition, failure to detect the presence of SARS-CoV-2 in an assay does not necessarily mean that the test is a ‘false negative’. It might simply indicate that the pathogen was below the lower limits of detection for this test; thus, all diagnostic tests should be locally validated to understand performance characteristics.

Few data are available to demonstrate the value of repeat patient testing to improve COVID-19 diagnosis. Apart from delay in diagnosis and patient inconvenience, repeat testing adds a further burden on constrained labour and reagent resources. Therefore, data to determine optimal testing policies are needed. In this report, we evaluated our experience with SARS-CoV-2 testing in order to investigate the added value of repeat patient testing to diagnose COVID-19.

## Methods

### Study design, setting and patients

Northwestern Memorial Hospital (NMH) is an 885-bed teaching hospital in Chicago, Illinois, USA. SARS-CoV-2 testing results from 1 March 2020 to 30 April 2020 were collected from the electronic medical record and exported to Enterprise Data Warehouse (EDW). The data include SARS-CoV-2 prevalence rates in Chicago during the study period, patient demographics, test name, specimen type and test results. Patients who had more than one sample tested, including nasopharyngeal (NP) swabs or bronchoalveolar lavage (BAL) samples, were included in the study. Each testing assay has a unique test code. The selection of assay was carried out automatically based on patient locations when an order was placed in accordance with existing laboratory protocols. Repeat testing was at the discretion of the treating physician. During the study period, only symptomatic patients were tested. Patient demographic information was removed before the data were analysed. Information on testing times, specimen type, assay used and test result was reviewed. All test results were reported within <24 h of specimen collection. Only test results for NP and BAL were included in the analysis. The study was exempt from the Institutional Research Board approval.

### SARS-CoV-2 testing assays used

During the study period, three SARS-CoV-2 testing assays, the CDC SARS CoV-2 RT-qPCR panel, Xpert Xpress CoV-2 (Cepheid, Inc., Sunnyvale, CA, USA) and ID NOW COVID-19 (Abbott Laboratories, Chicago, IL, USA), were used at our institution. Only test results for NP samples and BALs were included in the study. NP swabs collected in viral transport medium (VTM) were used for testing with the Xpert Xpress CoV-2 assay and the CDC SARS CoV-2 RT-qPCR panel. During the study period, VTM was purchased from various manufacturers, including Remel; Becton, Dickinson and Company; Handle, Trinity Biotech, Inc; and Hardy Diagnostics due to national resource constraints. All VTM was validated prior to use for all three assays. NP swabs were used for testing with ID NOW COVID-19. For the Xpert Xpress CoV-2 assay, results with a cycle threshold (*C*_t_) value <45 were called positive. For the CDC SARS CoV-2RT-qPCR panel, viral RNA was extracted from clinical specimens utilizing the QIAamp Viral RNA Minikit (Qiagen, Carlsbad, CA USA); reverse transcription and PCR (RT-qPCR) with the CDC 2019-nCoV RT-qPCR Diagnostic Panel were performed with N1 and N2 probes in SARS-CoV-2 and RP probes for sample quality control using QuantaStudio 5 (Thermo Fisher Scientific, Waltham, MA, USA) as described previously [1]. All specimens with an N1 probe (*C*_t_ less than or equal to 35 were considered positive. Indeterminate results were primarily generated by the CDC RT-qPCR panel and defined as RT-qPCR with a *C*_t_ value of N1 between 35 to 40 [1]. The *C*_t_ cutoff was determined based on the data generated by the assay verification study. Invalid results were caused by failure of the instrument, reagents, or internal controls.

## Results

### Results of repeat testing

From 1 March 2020 to 30 April 2020, the test positivity rate for SARS-CoV-2 in Chicago changed from 0 to 26.73 %, with a rate of 10.6 % by 7 March 2020 and a peak of 29.99 % on 9 April 2020 (https://www.chicago.gov/city/en/sites/covid-19/home/latest-data.html). During the study period, a total of 1445 patients were tested at our hospital more than once for SARS-CoV-2 (Table 1). Among them, 1204 (83.3 %) patients were tested twice, 181 (12.5 %) patients were tested 3 times and 60 (4.2 %) patients were tested 4 times or more. One patient was tested nine times. Clinicians were more likely to repeat initial negative tests, as 1189 of 1445 (82.3 %) repeat tests occurred in patients who were initially negative. Among these, the time between the initial negative test and the second test ranged from 0 to 1100 h (median: 60 h; IQR 25–75 : 18–231 h). The time between the second and third tests for these patients ranged from 0 to 968 h (median: 88 h; IQR 25–75 : 30–221 h), and between the third and fourth tests it ranged from 0 to 1031 h (median: 100 h; IQR 25–75 : 46–203 h). On average, clinicians were slower to repeat each subsequent test.

**Table 1.** Overview of patients who underwent repeat testing

Tests taken	No. of patients	% of patients
2	1204	83.3 %
3	181	12.5 %
4	42	2.9 %
5	12	0.8 %
6	3	0.2 %
7	2	0.1 %
9	1	0.1 %
**Total**	**1445**	**99.9 %**

The results for patients (*n*=1204) who were tested twice were further analysed (Table 2). The test results were concordant for 1036 (86.0 %) patients and discordant for 168 (14 %). Of the 1036 concordant results, 943 (91 %) tested negative, 89 (8.6 %) tested positive and 4 (0.4 %) tested indeterminate. Among the 168 discrepant results, 72 (42.9 %) patients tested negative on test 1 and positive on test 2; 13 (7.7 %) tested positive on test 1 and negative on test 2; 80(48 %) tested indeterminate on test 1 and positive (*n*=13) or negative (*n*=67) on test 2; and 3 (1.8 %) were originally positive (*n*=2) or negative (*n*=1) but indeterminate on test 2. Of note, 80 (95 %) of 84 initially indeterminate results yielded a definitive answer on the second test. Of the 72 who were tested twice with negative and then positive results, the median time between tests was 202 h. This finding could represent delayed clinician testing and detection of patients who were initially positive, but could also represent nosocomial acquisition of SARS-CoV-2; either way, this finding has significant infection control implications.

**Table 2.** Results for 1204 patients who were tested twice

Test 1	Test 2	No. of patients	% of category patients	% of total patients	Average hours between repeat tests
Positive	Positive	89	85.6	7.4	154
	Negative	13	12.5	1.1	176
	Indeterminate/invalid	2	1.9	0.2	132
	Total	104	100	8.7	N/A
Negative	Positive	72	7.1	6.0	202
	Negative	943	92.8	78.3	164
	Indeterminate/invalid	1	0.1	0.1	187
	Total	1016	100	84.4	N/A
Indeterminate/invalid	Positive	13	15.5	1.1	53
	Negative	67	79.8	5.6	62
	Indeterminate/invalid	4	4.8	0.3	47
	Total	84	100	7	N/A

The results for patients (*n*=181) tested three times are summarized in Table 3. For 144 (79.5 %) patients, all subsequent testing yielded the same results as the initial result. Thirty-seven (20.5 %) patients had at least one result that was different from the initial result. Among them, only three (1.7 %) initially negative patients tested positive by subsequent testing. Repeat testing resolved the indeterminate/invalid results for 7 (63.6 %) of 11 patients.

**Table 3.** Results for 181 patients who were tested three times

Test 1	Tests 2 and 3	No. of patients	% of category patients	% of total patients
Positive	Positive	17	68	9.4
	Negative	4	16	2.2
	Other	4	16	2.2
	Total	25	100	13.8
Negative	Positive	3	2.1	1.7
	Negative	127	87.6	70.2
	Other	15	10.3	8.3
	Total	145	100	80.2
Indeterminate/invalid	Positive	1	9.1	0.6
	Negative	6	54.5	3.3
	Other	4	36.4	2.2
	Total	11	100	6.1

Positive, consistent positive result after the first test; negative, consistent negative results after the first test; others, result combinations without a consistent result.

Table 4 summarizes the results for 60 patients tested 4 times or more. For 33 (55 %) patients, repeat testing yielded the same results as the initial ones, while 27 (45 %) patients had different results. None of patients tested negative initially tested positive consistently in the subsequent testing. Three patients with indeterminate/invalid results tested either positive or negative by repeat testing.

**Table 4.** Results for 60 patients tested four times or more

Test 1	All subsequent results	No. of patients	% of category	% of total patients
Positive	Positive	5	25	8.3
	Negative	0	0	0
	Other	15	75	25
	Total	20	100	33.3
Negative	Positive	0	0	0
	Negative	28	77.8	46.7
	Other	8	22.2	13.3
	Total	36	100	60
Indeterminate/invalid	Positive	1	25	1.7
	Negative	2	50	3.3
	Other	1	25	1.7
	Total	4	100	6.7

Positive, consistent positive result after the first test; negative, consistent negative results after the first test; others, result combinations without a consistent result.

### Repeat testing of different specimen types

Forty-one patients were tested for SARS-CoV-2 initially using an NP sample followed by a BAL sample (Table 5). Of these patients, the first tests by NP swab were conducted as follows: 33 performed with the CDC assay, 2 with IDNow and 6 with Xpert Xpress. This was followed by BAL as follows: 31 performed with CDC assay and 10 with Xpert Xpress. The test results for NP and BAL were in agreement for 39 (95.1 %) patients. One patient was positive by NP but negative by BAL. One patient was negative by NP but positive by BAL.

**Table 5.** NP testing followed by BAL testing

	NP-positive	NP-negative	Total
BAL-positive	16	1	17
BAL-negative	1	23	24
Total	17	24	41

### Initial testing using different platforms

Three SARS CoV-2 testing assays were used. The outcomes of repeat patient testing were analysed for each assay (Table 6). When the initial test was performed with the CDC SARS CoV-2 RT-qPCR panel (*n*=668, 46.2 %), repeat testing produced the same results as the initial ones for 542 (81.1 %) patients. Twenty-eight patients (4.3 %) tested negative initially but were positive in the subsequent test, while 13 (2 %) changed from positive to negative. Among the 85 patients with indeterminate/invalid results, repeat testing provided definitive results for 81 (95.3 %).

**Table 6.** Testing platforms

Test 1 assay	Result	Test 2 result		% of patients
CDC (*n*=668)	Positive (*n*=90)	Positive	78	11.7
		Negative	13	2.0
		Indeterminate/invalid	1	0.2
		Total	90	13.5
	Negative (*n*=485)	Positive	28	4.2
		Negative	460	68.9
		Indeterminate/invalid	5	0.7
		Total	493	73.8
	Indeterminate (*n*=85)	Positive	12	1.8
		Negative	69	10.3
		Indeterminate/invalid	4	0.6
		Total	85	12.7
Cepheid (*n*=421)	Positive (*n*=38)	Positive	31	7.4
		Negative	3	0.7
		Indeterminate/invalid	4	1
		Total	38	9.1
	Negative (*n*=382)	Positive	10	2.4
		Negative	371	88.1
		Indeterminate/invalid	1	0.2
		Total	382	90.7
	Invalid (*n*=1)	Positive	0	0
		Negative	1	0.2
		Indeterminate/invalid	0	0
		Total	1	0.2
ID Now (*n*=356)	Positive (*n*=19)	Positive	18	5.1
		Negative	1	0.3
		Indeterminate/invalid	0	0
		Total	19	5.4
	Negative (*n*=328)	Positive	39	11
		Negative	288	80.9
		Indeterminate/invalid	1	0.3
		Total	328	92.2
	Invalid (*n*=9)	Positive	4	1.1
		Negative	1	1.1
		Indeterminate/invalid	1	.03
		Total	9	2.5

When the initial test was performed with the Xpert Xpress CoV-2 (*n*=421, 29.1 %), repeat testing produced the same results as the initial ones for 402 (95.5 %) patients. Test 2 produced positive results for 10 (2.4 %) patients who were negative on test 1 and negative results for 3 (0.7 %) patients who were positive on test 1. Only one patient had an indeterminate/invalid result on test 1. The patient later tested negative on test 2.

Three hundred and fifty-six (24.6 %) patients were tested using ID NOW initially. Test 2 produced the same results as test 1 for 307 (86.2 %) patients. Thirty-nine (11%) patients tested negative initially but were positive in the subsequent test. Only one (0.3 %) tested positive on test 1 but negative on test 2. For the nine patients with indeterminate/invalid results, repeat testing resolved the indeterminate/invalid results for eight (88.9 %).

## Discussion

Our experience with repeat patient testing demonstrated that the majority of repeat testing was performed when initial test results were negative. As shown in Tables 2 and 3, more than 80 % of repeat testing was performed for patients who had a negative first test result. A small percentage of repeat testing, 5.6 and 6.1 %, was for patients who had indeterminate/invalid initial test results. Approximately 10 % of repeat testing was performed for patients who initially tested positive for reasons such as discharge planning and following disease course or treatment outcomes.

The yield for using repeat testing to identify false-negative patients was low. As our study revealed, when test 1 produced a negative result, only 6 % of patients tested positive in the second test. The yield reduced to 1.7 % after the third test and further reduced to 0 % after the fourth test. COVID-19 patients with typical clinical COVID-19 features and evidence of pneumonia on computed tomography (CT) imaging but a negative NAA test result have been reported [7]. Investigation of SARS-CoV-2 viral load in upper respiratory specimens of infected patients showed that viral load progressively decreases over time and can drop below the level of detection after 8 days from symptom onset [8, 9]. If patient admission occurred at the later stage of the disease process, the negative results may represent true negative results. In this situation, clinical and radiographic findings should be combined with epidemiological history to arrive at a likely diagnosis. Serological assays can also be used as supplemental tests to facilitate the diagnosis of difficult cases, although this can be confounded now that vaccines are available and as, over time, reinfection becomes possible. Positive patients missed by test 1 but detected by repeat testing were infrequent. Considering the limited yield of repeat testing and the shortage of sample collection devices and testing reagents, testing patients more than two times with NAA should be discouraged unless clinical suspicion is high.

When comparing the results produced by the three assays, the CDC SARS CoV-2 RT-qPCR panel, Xpert Xpress CoV-2 and ID NOW COVID-19, the ID NOW COVID-19 assay was associated with the highest number of false-negative initial results, with 11 % for ID NOW and 4.3 and 2.4 % for the CDC SARS CoV-2 RT-qPCR panel and the Xpert Xpress CoV-2, respectively. This can likely be explained by the difference in test performance. The reported limits of detection for the three assays are 200, 100 and 20 000 copies ml^−1^ [1, 10], respectively; thus, the limit of detection for ID NOW was 200 times higher than that of Xpert Xpress and the CDC panel. When test 1 was performed with ID NOW and a more sensitive assay was used for the repeat test, more positive results were produced. The conversion of negative to positive observed for the CDC panel and Xpert Xpress was presumably caused by variable sample collection or changes in viral load associated with the timing of testing.

Our study demonstrated that sending repeat testing was helpful for resolving indeterminate/invalid results. As indicated in Table 2, more than 90 % of initial indeterminate/invalid results were resolved by repeat tests, which produced either positive or negative results. While ID NOW and Xpert Xpress CoV-2 generated a few invalid results, 13 % of results produced by the CDC assay (22/189) were in the indeterminate range. Because the methodologies, targets and limits of detection differ for each assay, using ID NOW and Cepheid provided an alternative solution to resolve indeterminate results from the CDC panel.

Studies have demonstrated that in general lower respiratory tract samples have higher viral loads and better yield of detection than upper respiratory tract samples [9, 11], and in severe or progressive disease, patients with negative initial samples, particularly from the upper respiratory tract, should be given repeat testing using a lower respiratory specimen. Huang *et al*. compared the detection of SARS-CoV-2 in various samples of 16 critically ill patients and found that NP samples from 13 patients were positive while sputum/ET samples were positive from all 16 patients. Higher yield of viral detection in the lower respiratory samples was not observed in our study. No major differences were seen when testing BAL and NP samples from the same patient, indicating that sending repeated testing from the lower respiratory tract when testing of the upper respiratory tract sample was negative may not change the test result. Further study is necessary to clarify whether the variation in yield of viral testing is associated with the sampling or the disease process.

Our study was conducted early in the first wave of the COVID-19 pandemic when the prevalence of disease was rising rapidly in Chicago. During this relatively short time frame, it was very unlikely that infected patients were reinfected. Further, all decisions for repeat testing were based on whether clinicians trusted the initial results, or were based on subsequent clinical presentations. Additionally, at the time tests were sent, no repeat testing was conducted for pre-procedure or preoperative testing, as is now mandated in certain public health guidance, and no testing was sent because clinicians were concerned about prolonged shedding, because this phenomenon was not yet fully recognized. Thus, we think that the number of repeat tests that led to the ‘correct’ clinical answer that clinicians trusted is well represented by these data. We think this study reassures clinicians that the value of repeat testing is low, and is very low after the second test. This study quantitates the value of repeat testing for clinicians and helps inform infection prevention efforts.

Our study had several limitations. First, data analysis was performed with aggregate data. The level of granularity is limited. Second, we described the use of three different tests. Each test has different analytical performance. Ideally, analysis of repeat tests performed with the same assay would provide more accurate results. However, due to testing reagent shortage, patient specimens were tested with mixed assays. Our study results apply to the true clinical situation. Third, the time intervals between repeat tests are important variables that could impact on the interpretation of the results. Limited time interval data were evaluated. An association between time interval and repeat test outcome was not identified.

In conclusion, a review of our experience with repeat patient testing for SARS-CoV-2 with NAA assays showed that the yield from using repeat testing to identify false-negative patients was low. Repeat testing was best used for resolving indeterminate/invalid results.
